# UPLC-QTOF/MS Analysis of Alkaloids in Traditional Processed *Coptis chinensis* Franch.

**DOI:** 10.1155/2012/942384

**Published:** 2012-12-13

**Authors:** Xue Jiang, Lin-Fang Huang, La-Bin Wu, Zeng-Hui Wang, Shi-Lin Chen

**Affiliations:** Institute of Medicinal Plant Development, Chinese Academy of Medical Sciences and Peking Union Medical College, Beijing 100193, China

## Abstract

The processing technology employed in traditional Chinese medicine (TCM) is significant and distinct. Meanwhile, the processed *Coptis chinensis* Franch. are significant in clinic based on clinical practice and literature. The current study used ultraperformance liquid chromatography method (UPLC) coupled with quadrupole time of flight mass spectrometry (qTOF/MS) and Marklynx software to analyze the chemical profiles of crude and processed *C. chinensis* Franch. 13 compounds in these samples are identified, including 3 compounds that are detected in *C. chinensis* Franch. for the first time. Moreover, the results of the experiment show significant chemical differences between crude and processed *C. chinensis* Franch. with principal component analysis (PCA). The obvious separation in PCA confirms the traditional processing theory in TCM.

## 1. Introduction 

Traditional processing technology (Paozhi technology) is a significant part of traditional Chinese medicine (TCM). Employing the correct processing technology is necessary in manufacturing clinical decoction pieces. According to the restrictions in the science of TCM, any decoction piece must be processed by certain methods before it can be used in clinical practice. Processing technology was developed almost 5000 years ago, along with a number of processing technology theories and methods. Briefly, processing technology plays three main roles in TCM. First, it decreases the toxicity of some TCMs. Some crude TCMs have high toxicity and various side effects; thus, numerous processing technologies have been developed to remove the toxicity and decrease the side effects. Second, processing technology increases the pharmacological effects. Third, processing technology creates new drugs that have new pharmacological effects as a pharmaceutical method. The reason for developing this is when faced with complex human diseases, crude TCMs are sometimes unable to satisfy the need in clinical practice due to the limitations posed by herbal medicine. Thus, the ancient Chinese created processing technology to produce abundant new drugs. In this paper, we investigate the five kinds of processed *Coptis chinensis *Franch. in chemical profiles. 

Processing technology was developed via clinical practice experience and processing technology theories. The processing technology theories were based on the Traditional Five Elements Theory and the Yin-Yang Theory from ancient China. The five kinds of processed *C. chinensis *Franch. are originally based on the important theories in processing technology: Processing Synergy Theory (PST) or Cong Zhi and Processing Antagonism Theory (PAT) or Fan Zhi. Processed *C. chinensis *Franch. with rice wine (Jiu HL), with *Zingiber officinales *Rosc. (Jiang HL), and with *Euodia rutaecarpa *(Juss.) Benth (Yu HL) belong to PAT, whereas those processed with vinegar (Cu HL) and with salt (Yan HL) belong to PST. Such classification bases on the PST/PAT theory, the contribution of *C. chinensis *Franch. and their auxiliary materials. These processed products have different pharmacological effect in clinic according to the traditional literatures. The rice wine processed *C. chinensis* is specially used for the treatment of inflammation in head. The ginger processed *C. chinensis* is focusing on the treatment in stomach. The *Euodia rutaecarpa *(Juss.) Benth processed *C. chinensis* is specially used for the treatment of humidity/heat in liver and gall [1]. The vinegar and salt processed *C. chinensis* is used for extreme heat symptoms, which is totally different from the earlier three based on the TCM theory. The products of processed *C. chinensis *Franch. presented unique pharmacological effects in mice experiments [2–6]. In our investigation, ultraperformance liquid chromatography method (UPLC) coupled with quadrupole time of flight mass spectrometry (UPLC-qTOF/MS) is applied to explore the material basis in these processed products with different bioactivities. 

 The major active constituents in *C. chinensis* are alkaloids, including berberine, jatrorrhizine, coptisine, and palmatine [7]. The berberine, present at about 10% in *C. chinensis* Franch., has strong antibacterial bioactivities on *Shigella dysenteriae*, staphylococci, and streptococci and has been used for of dysentery [8], which is also one of the most popular drugs in academia. Palmatine had extensive pharmacological actions including antibacterial activity such as *Escherichia coli,* and *Staphylococcus aureus* [9], anti-inflammation [10], and anticancer effect [11]. Therefore, the analysis on alkaloids in* C. chinensis *Franch. is significant, which decides the therapeutic effects. Chen et al. has investigated *C. chinensis *Franch. from different locations based on high-performance liquid chromatography-electrospray ionization-time-off light mass spectrometry (HPLC-ESI-TOF-MS), ultraperformance liquid chromatography/photodiode array detector (UPLC/PDA), and UPLC-MS/MS for authentication and quality evaluation [12]. And these results indicated that both UPLC-PDA and UPLC-MS/MS methods were simple, sensitive, and reliable for the determination of alkaloids in *C. chinensis *Franch. In this paper, it is the first time to investigate the composition of main compounds in crude and processed *C. chinensis *Franch. by UPLC-qTOF/MS. 

## 2. Materials and Methods

### 2.1. Chemicals, Reagents, and Materials

Acetonitrile and formic acid were purchased from Fisher Scientific Co. (MA, USA). Ammonium acetate was purchased from Xilong Company (Shanxi, China). All aqueous solutions were prepared with ultrapure water produced by Milli-Q system (18.2 MΩ, Millipore, Ma, USA). Berberine, palmatine, coptisine, epiberberine, and jatrorrhizine standards were purchased from Must Company (Sichuan, China). *C. chinensis *Franch., *Z. officinales *Rosc., and *E. rutaecarpa *(Juss.) Benth were purchased from Sichuan Chinese Herbs Corporation (Sichuan, China). The botanical materials were identified by Professor Chen Shilin, and the voucher specimens were deposited in the Institute of Medicinal Plant Development, Chinese Academy of Medical Sciences, Beijing, China. 

### 2.2. Instrumentation and Chromatographic Conditions

UPLC-qTOF/MS analysis was performed using a qTOF Synapt G2 HDMS system (Waters, Pittsburgh, PA, USA) equipped with an ESI source operated in the positive ion mode. N_2_ was used as the desolvation gas. The desolvation temperature was set at 450°C with a flow rate of 800 L/h and a source temperature of 120°C. The capillary and cone voltages were set to 2500 and 40 V. Data were collected between 50 Da and 1200 Da, with a scan time of 0.1 s and interscan delay of 0.01 s over an analysis time of 16 min. 

### 2.3. Preparation of the Sample Solution

*C. chinensis *Franch. samples were cut into 1.5 mm slices and then processed according to the methods described in China pharmacopoeia [1]. Then, the samples were processed at a temperature of 160°C and dried in an oven after mixed with auxiliary material consisting of rice wine (20% w/w), salt (10% w/w), vinegar (20% w/w), *E. rutaecarpa *(Juss.) Benth (10% w/w), and *Z. officinale *Rosc. (10% w/w) [13]. All samples were milled into powder and dried at 30°C in oven until they attained constant weight. A total of 0.150 g powder samples were dissolved in a methanol-sulfuric acid (100 : 3) solution. Next, the samples were extracted by ultrasonic cleaner for 30 min before a 15 min water bath (60°C). The sample solutions were subsequently filtered through a 0.22 *μ*m membrane and then injected into the UPLC-qTOF system for analysis. 

### 2.4. Preparation of Standard Solution

The standard stock solutions of berberine (0.12 mg/mL), palmatine (0.11 mg/mL), berberrubine (0.118 mg/mL), coptisine (0.115 mg/mL), epiberberine (0.107 mg/mL), and jatrorrhizine (0.115 mg/mL) were prepared in methanol and stored at −4°C. The solutions were brought to room temperature and filtered through a 0.22 *μ*m membrane filter before injection. 

### 2.5. Data Analysis

The UPLC-qTOF/MS data of crude *C. chinensis *Franch. and processed *C. chinensis *Franch. samples were analyzed to identify discriminant variables. The peak finding, peak alignment, and peak filtering of ES+ raw data were carried out with Markerlynx applications manager version 4.1. The parameters used were within the retention time of 0–10 min, and mass range 50–1200 Da, mass tolerance 0.02 Da. Noise elimination level was set at 6.00, and minimum intensity was set to 15% of base peak intensity. 

## 3. Results and Discussion 

### 3.1. UPLC Method Development

To produce a better chromatogram in UPLC-qTOF, the UPLC method was developed with consideration for such factors as mobile phases, modifiers, and flow rates. Methanol and acetonitrile were tested with different ratios, linear gradients, and flow rates of the mobile phase (0.1, 0.2, and 0.25 mL/min). The modifiers, such as formic acid, ammonium acetate, sodium dodecyl sulfate (SDS), phosphoric acid, and diethylamine, were all detected in the present experiment. As a result, water containing 1% formic acid and 1% ammonium acetate (A)—acetonitrile (B) with a flow rate of 0.25 mL/min was chosen as the optimum chromatographic condition with a linear gradient (0–10 min, 80%–70% A) at room temperature. 

### 3.2. UPLC-qTOF/MS Chemical Analysis

**Figure 1:**
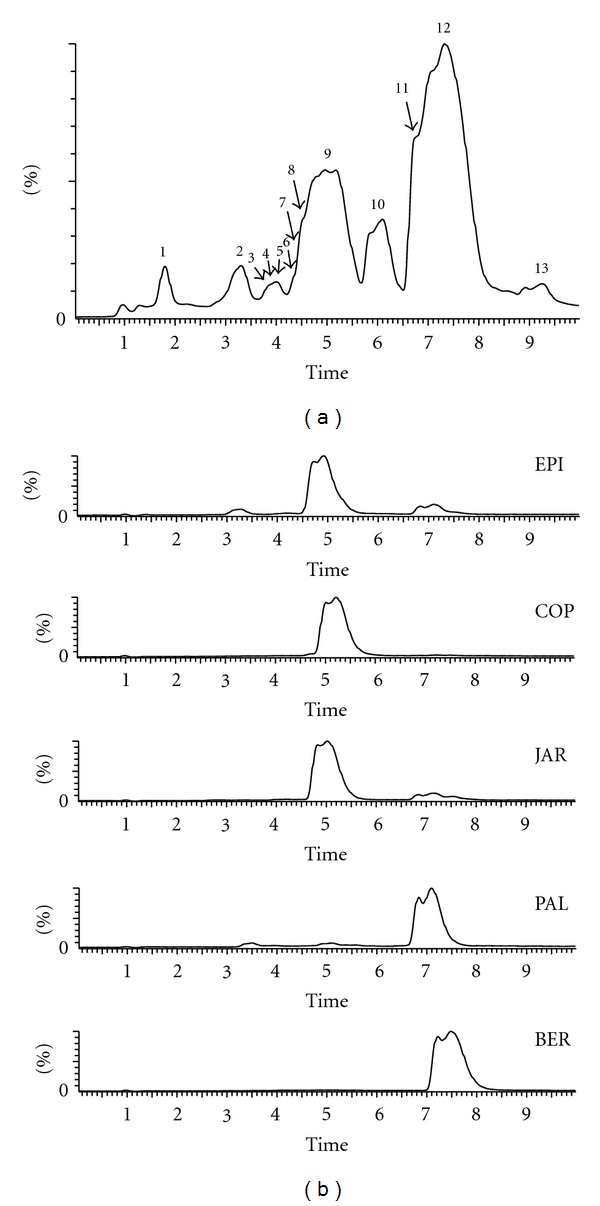
(a) The representative chromatogram of *C. chinensis *Franch. by UPLC-qTOF/MS (the numbers of peaks are same with the identification in Table 1). (b) The chromatograms of standard compounds (EPI: epiberberine; COP: coptisine; JAR: jatrorrhizine; PAL: palmatine; BER: berberine).

Figure 1(a) presents the representative chromatogram of *C. chinensis *Franch. by UPLC-qTOF/MS. Figure 1(b) shows the five standard compounds chromatograms.  Table 1 shows 13 compounds in the chromatograms that have been identified based on [M+H]^+^ *m/z* and retention time analysis by the database and references. In the experiments, the chromatograms of the samples were analyzed using the Marklynx software. More than 3000 markers (differences) were detected to be present among the crude and processed *C. chinensis *Franch. 

Alkaloids comprise the main compounds in *C. chinensis *Franch., including berberine, palmatine, coptisine, epiberberine, jatrorrhizine, columbamine, and magnoflorine. Berberine, palmatine, coptisine, epiberberine, and jatrorrhizine are the five main alkaloids in *C. chinensis *Franch., with their contents range from 5% to 10% in total [1]. Among them, berberine, coptisine, epiberberine, jatrorrhizine are the chemical indicators in evaluating the quality of *C. chinensis *Franch. in China pharmacopoeia [1]. Although the compounds, like magnoflorine and columbamine, with very lower contents, are difficult to identify in chromatographs [20], they help explain the pharmacological effects of *C. chinensis *Franch. Therefore, identifying these compounds is significant. In our study, the UPLC-qTOF/MS provides the information of 13 compounds in *C. chinensis *Franch. 

 Except lincangenine/stephabine, lycoranine B, and dihydrochelerythrine, all these identified compounds have been reported in *C. chinensis *Franch. in [13–15, 21] with significant bioactivities. And all the identified compounds are protoberberine alkaloids with the similar structures chemically. Lincangenine/stephabine and lycoranine B have been isolated from *Stephania suberosa* and *Lycoris radiata*, respectively [16, 18]. And dihydrochelerythrine could be transformed for berberine chemically [19]. These references show a higher possibility for the existence of lincangenine/stephabine, lycoranine B, and dihydrochelerythrine in *C. chinensis *Franch. And they are the first time to be reported in *C. chinensis *Franch. Lincangenine and stephabine are isomeric compounds, and they both have the possibility to exist in *C. chinensis *Franch. Jatrorrhizine/columbamine and thalifendine/berberrubine are the isomeric compounds, respectively. And they could all exist in *C. chinensis *Franch. because they could transform into each other with biosynthesized way [22]. 

### 3.3. Dihydrochelerythrine Contained in Crude and Processed *C. chinensis *Franch.

**Figure 2:**
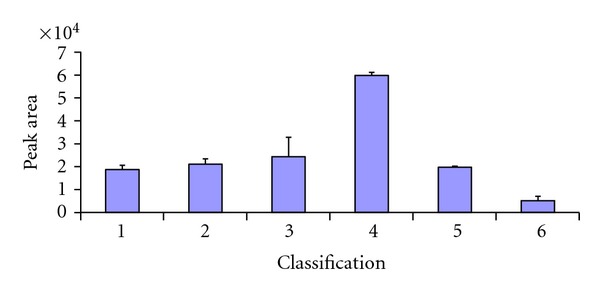
(1) Jiang HL, (2) Yu HL, (3) Jiu HL, (4) Cu HL, (5) Yan HL, (6) crude *C. chinensis *Franch. The relative content of dihydrochelerythrine in crude and processed *C. chinensis Franch* (*N* = 3).

Dihydrochelerythrine was detected in this study.  Figure 2 presents the relative content (based on the peak area) in crude and processed *C. chinensis *Franch. It shows that the relative content of dihydrochelerythrine in crude *C. chinensis *Franch. is very lower than other samples. And the relative content of dihydrochelerythrine in Cu HL is the highest. It is possible that the auxiliary material or heating process could enhance the transformation of dihydrochelerythrine from berberine. In our previous study, the transformation of berberine to berberrubine, palmatine to palmatine, could be increased with an acidic condition. And the result in this study confirm the general rule of transformation with protoberberine alkaloids in processed *C. chinensis *Franch. that acidic condition in processing could enhance the protoberberine transformation. Dihydrochelerythrine has been reported with many pharmacological activities, such as antiparasitic and antitumor effects [23, 24]. And the reason for increasing the content of dihydrochelerythrine in processed *C. chinensis *Franch. would be explored further. 

### 3.4. Confirmation of TCM Processing Theories with Crude and Processed *C. chinensis *Franch.

**Figure 3:**
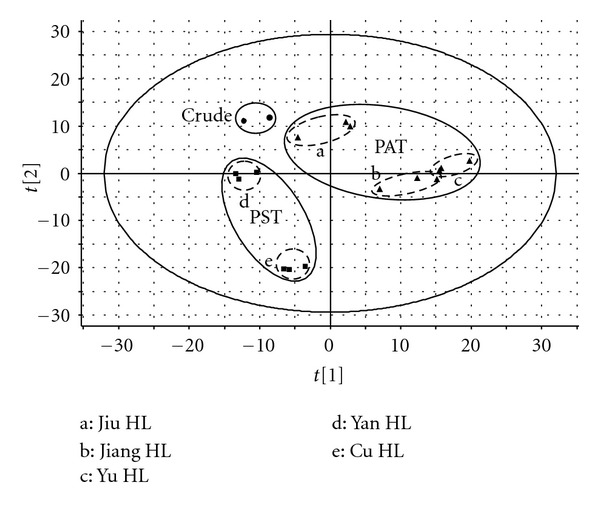
PCA of crude and processed *C. chinensis *Franch.

There are 5 kinds of processed *C. chinensis* Franch.: Jiu HL, Jiang HL, Yu HL, Cu HL, and Yan HL. Crude *C. chinensis* Franch. and 5 different processed products were analyzed by PCA. Figure 3 shows the differences of crude and processed *C. chinensis* Franch. In the PCA, crude and 5 processed *C. chinensis* Franch. have been separated clearly. Jiu HL, Jiang HL, and Yu HL (PAT) are clustered into one side, and Cu HL and Yan HL (PST) are clustered into the other side. Among them, Jiu HL and Jiang HL are positioned in positive region; crude, Cu HL and Yan HL are positioned in the negative region, while Jiu HL group is in the middle of these two regions. 

 According to the TCM theories, all the TCMs could be grouped into 4 classifications, which are cold, heat, warm, and cool. And *C. chinensis* Franch. is in the classification of “cold,” and it belongs to the extreme cold level. This contribution of *C. chinensis* Franch. could lead to some side-effects if patients take it for a long period of time or some patients with special physiques take it. Therefore, the contribution of *C. chinensis* Franch. should be modified to fit the specific requirement in clinic. On one side, *C. chinensis* Franch. should be adjusted into a little “warm” to eliminate the side-effect of the extreme cold character. On the other side, *C. chinensis* Franch. could be modified into more “cold” to meet the extremely heat syndrome in clinic. The former one should be treated as the method of PAT, while the latter one should be treated as the method of PST. Among the PAT, Jiang HL and Yu HL could transform more of *C. chinensis* Franch. into the “warm” side, while Jiu HL could transform less compared with Jiang HL and Yu HL. And this could be confirmed in the PCA that Jiang HL and Yu HL are in the positive region while Jiu HL is in the middle of the two regions. And crude, Cu HL and Yan HL belong to the contribution of “cold” with the positions of negative region in PCA. Thus, the result of PCA conforms with the traditional processing theories and illustrates the methods of PAT and PST. 

 Our experiment shows the differences in the 5 processed *C. chinensis* Franch. which can be identified by UPLC-qTOF and analyzed by Markerlynx software. And this method could demonstrate the TCM theories markedly. This is the first time to elucidate TCM processing theories by modern technology. 

## 4. Conclusion 

Forms of processed *C. chinensis *Franch. have been recorded in the long history of TCM. Their curative effects have also been verified in clinical setting. The chemical analysis of processed *C. chinensis *Franch. via UPLC-qTOF/MS demonstrates significant differences. Such information demonstrates the significance of processed *C. chinensis *Franch. as well as of PAT and PST drugs. The processing technology helps create new pharmacological drugs that possess distinctive clinical effects. This result, therefore, is a new finding in traditional alternative medicine. 

## Figures and Tables

**Table 1:** Identified compounds in *C. chinensis* Franch. by UPLC-QTOF/MS.

Peak no.	*t*_*R*_ (min)	Identified compounds	Molecular formula	[M+H]^+^ *m*/*z*			Reference
				Mean measured mass (Da)	Theoretical exact mass (Da)	Mass error (ppm)	
1	1.79	Magnoflorine	C_20_H_23_NO_4_	342.1703	342.1705	−0.6	[13–15]
2	3.32	Groenlandicine	C_19_H_15_NO_4_	322.1064	322.1079	−4.7	[14]
3	3.81	Berberastine	C_20_H_17_NO_5_	352.1184	352.1185	−0.3	[14]
4	3.71	Lincangenine/stephabine	C_21_H_21_NO_5_	368.1506	368.1498	2.2	[16]
5	4.02	Demethyleneberberine	C_19_H_17_NO_4_	324.1220	324.1236	−4.9	[17]
6	4.32	Lycoranine B	C_18_H_13_NO_4_	308.0903	308.0923	−6.5	[18]
7	4.50	Jatrorrhizine/columbamine	C_20_H_19_NO_4_	338.1386	338.1392	−1.8	[13, 14]
8	4.66	Epiberberine	C_20_H_17_NO_4_	336.1228	336.1236	−2.4	[13, 14]
9	4.95	Coptisine	C_19_H_13_NO_4_	320.0915	320.0923	−2.5	[13, 14]
10	5.81	Thalifendine/berberrubine	C_18_H_15_NO_4_	322.1070	322.1079	−2.8	[14]
11	6.68	Palmatine	C_21_H_21_NO_4_	352.1556	352.1549	2.0	[13, 14]
12	7.01	Berberine	C_20_H_17_NO_4_	336.1236	336.1236	0	[13, 14]
13	8.90	Dihydrochelerythrine	C_21_H_19_NO_4_	350.1394	350.1392	0.6	[19]

## Abbreviations

UPLC-qTOF/MS: Ultra-performance liquid chromatography-quadrupole time of flight mass spectrometry
PCA: Principal component analysis
TCM: Traditional Chinese medicine
PST: Processing synergy theory
PAT: Processing antagonism theory
Jiu HL: Processed *C. chinensis* Franch. with rice wine
Jiang HL: Processed *C. chinensis* Franch. with *Zingiber officinales* Rosc.
Yu HL: Processed *C. chinensis* Franch. with *Euodia rutaecarpa* (Juss.) Benth
Cu HL: Processed *C. chinensis* Franch. with vinegar
Yan HL: Processed *C. chinensis* Franch. with salt.
